# Change in diaphragmatic morphology in single-lung transplant recipients: a computed tomographic study

**DOI:** 10.3389/fphys.2023.1220463

**Published:** 2023-09-26

**Authors:** François Touchon, Julien Bermudez, Paul Habert, Fabienne Bregeon, Pascal Alexandre Thomas, Martine Reynaud-Gaubert, Benjamin Coiffard

**Affiliations:** ^1^ APHM, Department of Respiratory Medicine and Lung Transplantation, Aix Marseille University, Marseille, France; ^2^ APHM, Department of Radiology, Aix-Marseille University, Marseille, France; ^3^ LIIE, Aix Marseille University, Marseille, France; ^4^ CERIMED, Aix Marseille University, Marseille, France; ^5^ APHM, Pulmonary Function Testing Laboratory, Aix-Marseille University, Marseille, France; ^6^ APHM, Department of Thoracic Surgery, Aix-Marseille University, Marseille, France

**Keywords:** diaphragm, lung diseases, respiratory physiological phenomena, musculoskeletal physiological phenomena, lung transplantation, tomography, X-ray computed, transplant recipients

## Abstract

**Introduction:** The influence of lung disease on the diaphragm has been poorly studied. The study aimed to evaluate the diaphragm morphology (height and thickness) in single-lung transplantation (SLTx), using computed tomography (CT), by assessing the evolution of the hemidiaphragm of the transplanted and the native side.

**Methods:** Patients who underwent single lung transplantation in our center (Marseille, France) between January 2009 and January 2022 were retrospectively included. Thoracic or abdominal CT scans performed before and the closest to and at least 3 months after the surgery were used to measure the diaphragm crus thickness and the diaphragm dome height.

**Results:** 31 patients mainly transplanted for emphysema or pulmonary fibrosis were included. We demonstrated a significant increase in diaphragm crus thickness on the side of the transplanted lung, with an estimated difference of + 1.25 mm, *p* = <0.001, at the level of the celiac artery, and + 0.90 mm, *p* < 0.001, at the level of the L1 vertebra while no significant difference was observed on the side of the native lung. We showed a significant reduction in the diaphragm height after SLTx on the transplanted side (−1.20 cm, *p* = 0.05), while no change on the native side (+0.02 cm, *p* = 0.88).

**Conclusion:** After a SLTx, diaphragmatic morphology significantly changed on the transplanted lung, while remaining altered on the native lung. These results highlights that an impaired lung may have a negative impact on its diaphragm. Replacement with a healthy lung can promote the recovery of the diaphragm to its anatomical morphology, reinforcing the close relationship between these two organs.

## 1 Introduction

The diaphragm is the principal muscle of respiration. Because of his anatomical position, the morphological and functional evaluation of the diaphragm is not easy. Diaphragmatic dysfunction is an underdiagnosed cause of respiratory impairment. The relationship between the lung and the diaphragm is relatively unknown. In particular, the influence of lung disease on the diaphragm has been poorly studied.

Computed tomography (CT) allows a complete assessment of the diaphragm morphology, with an excellent intra- and inter-observer agreement (Ufuk et al., 2019), with satisfactory 3D reconstruction obtained (Pettiaux et al., 1997). Given its availability and ease of use, several studies have focused on the assessment of the diaphragm thanks to CT, particularly in patients undergoing chronic respiratory diseases. Using 3D reconstruction, diaphragmatic morphology has been studied in chronic obstructive pulmonary disease (COPD), where the length and surface of the diaphragm are reduced, compared to healthy subjects (Cassart et al., 1997). A decrement in the diaphragm CT density and dome height was associated with COPD severity (Donovan et al., 2021). Similarly, CT has been investigated in intensive care units, where mechanically ventilated patients (Lee et al., 2016) and patients with sepsis (Jung et al., 2014), experienced diaphragmatic atrophy. These data are promising, but more information is necessary to better define the relationship between the diaphragm and a diseased lung.

Lung transplantation (LTx), by replacing a diseased lung with a healthy one, is an interesting model to evaluate the evolution of the diaphragmatic morphology before and after transplantation in the context of lung disease. Even more appealingly, single lung transplantation (SLTx) allows a healthy and a diseased lung to coexist in the same patient, along with their respective hemidiaphragm. Only one work has investigated the morphology of the diaphragm of patients undergoing SLTx for emphysema to healthy subjects. Using CT 3D reconstruction, the total diaphragm surface area and surface area of the diaphragmatic dome remain smaller in transplant recipients (Cassart et al., 1999). However, the diaphragm shape and thickness have not been assessed in this study.

This study aimed to evaluate the diaphragm morphology (height and thickness) in single-lung transplant patients, using CT, by assessing the evolution of the hemidiaphragm of the transplanted and the native side.

## 2 Methods

### 2.1 Study population

Patients who underwent SLTx in our lung transplant center (Marseille, France) between January 2009 and January 2022, were included in this study. We retrospectively analyzed the medical records, including clinical, biological, functional, and radiological data. The Institutional Review Board of the French learned society for respiratory medicine -Société de Pneumologie de Langue Française-approved the study protocol (CEPRO 2022-038bis) and a notice of information and non-objection was given to all participants. Exclusion criteria were as follows: age <18 years, presence of pneumothorax, pneumomediastinum, missing CT data (death before CT analyzing, diaphragm not visible, blurred image not allowing a correct measurement). The following characteristics of the patients were collected: age, sex, body mass index, lung transplant side, lung transplant indication, duration of mechanical ventilation, duration of intensive care unit stay, and duration of hospitalization.

### 2.2 Diaphragm morphology assessment

We collected data from CT performed before and the closest to the LTx and at least 3 months after LTx. Thoracic or abdominal or thoracoabdominal CT were used to fully visualize the diaphragm. Images were reviewed in soft tissue windows in axial and coronal planes. In the axial plane, we assessed the minimal diaphragm crus thickness at the level of the origin of the celiac artery (Celiac min, Supplementary Figure S1A). In the coronal plane, we recorded diaphragm thickness along the anterior part of the L1 vertebral body at mid-level (L1 mid, Supplementary Figure S1B). These measurements were chosen in line with the results of a retrospective study investigating the effectiveness of CT compared to fluoroscopy in the diagnosis of hemidiaphragmatic paralysis. Celiac min and L1 mid had the best diagnosis performance to identify paralysis (Sukkasem et al., 2017). We measured the diaphragmatic height (Diaph height, Supplementary Figure S1C), defined as the length of the line from the highest point of the diaphragmatic dome to its perpendicular intersection with a line through the upper pole of L1. We assume that Diaph height may be useful in the assessment of diaphragmatic morphology, an increased Diaph height being an equivalent of an elevated hemidiaphragm on chest radiography.

### 2.3 Data collected

Clinical and biological data were retrospectively collected as follows: serum albumin level, routing lung function tests (forced vital capacity, FVC; forced expiratory volume in the first second, FEV1; FEV1/FVC ratio, total lung capacity, TLC) 6-min walk test (6MWT), presence of systemic corticosteroids and its dosage. We also collected whether the patient required high-dose bolus systemic corticosteroids after transplantation and their dosage.

We calculated the tracheal sphericity index (Supporting Material) by Supporting Material = x^2/y^2, with x: coronal axis tracheal diameter and y: sagittal axis tracheal diameter. Measurements were made on the same CT used to measure the diaphragm, at the level of the junction between the left subclavian artery and the aorta. In full inspiration, the trachea adopts an oval shape, with a Supporting Material that tends to deviate from 1, while in expiration, the trachea becomes rounder, with an Supporting Material close to 1.

### 2.4 Study outcomes

The primary outcome was the change in diaphragmatic morphology, i.e., Celiac min, L1 mid, and Diaph height, before and after SLTx, on both transplanted and native lungs. Secondary outcomes were the evolution of clinical data, albumin serum level, lung function tests, and 6MWT, before and after SLTx. Finally, we assessed the correlation between the diaphragmatic morphology on CT and the lung function tests.

### 2.5 Data analysis

A descriptive analysis was performed on the overall population. Continuous variables were expressed as medians and interquartile or means and standard deviations according to the distribution (Shapiro-Wilk test), and qualitative variables were expressed as numbers and percentages. Qualitative parameters were compared using Chi-2 tests. Quantitative parameters were compared using the Wilcoxon test. Paired data (pre-, and post-SLTx) have been compared using the Wilcoxon signed-rank test.

Correlation tests of the diaphragmatic morphology (Celiac min, L1 mid, Diaph height) on CT and the lung function test parameters (FEV1, FVC, FEV1/FVC, TLC, 6 MWT) were performed using the Spearman test.

All tests were two-tailed. A *p*-value of less than 0.05 was considered significant. Analysis was performed using R software version 4.2.1 (2022-06-23) (R Core Team (2018)). R: A language and environment for statistical computing. R Foundation for Statistical Computing, Vienna, Austria. URL https://www.R-project.org/).

## 3 Results

### 3.1 Baseline characteristics

During the study period, 42 patients underwent SLTx. A total of 31 patients were included in the study. Data were missing for 11 patients: 5 patients were dead before they could perform CT after transplantation, 4 CT were too blurred to be analyzed and 2 CT did not allow a complete assessment of the diaphragm.

The baseline characteristics of the included patients are described in Table 1. The mean age of participants was 61 ± 4 years and 74% were men. Most of SLTx performed were on the right lung (74%). The most common indication was pulmonary fibrosis (64%). Among fibrosis patients, 4 (20%) were progressive fibrosing interstitial lung diseases, and 16 (80%) were idiopathic pulmonary fibrosis, of which 5 were associated with genetic variants. Left SLTx indication was mostly emphysema (*p* = 0.006). All patients who underwent transplantation for chronic lung allograft dysfunction were bronchiolitis obliterans syndrome type. Most participants had a preserved muscular status, with low physical deconditioning (mean 6 MWT distance of 356 m) and no hypoalbuminemia (mean serum level of 37.8 mg/L). 15 (48%) patients received systemic corticosteroids, at relatively low doses, most posology being <0.25 mg/kg/day. Lung function tests were classically altered, mostly with a restrictive pattern, given the proportion of participants with pulmonary fibrosis in this study. There was a wide range in duration of mechanical ventilation (from 1 to 23 days), duration of ICU stays (from 5 to 28 days), and duration of hospitalization (28–71 days). CT were performed with proper inspiration, with median tracheal Supporting Material being 0.76.

**TABLE 1:** Baseline of the patient characteristics. SLTx: single-lung transplantation, CLAD: chronic lung allograft dysfunction, FEV1: forced expiratory volume in the first second, FVC: forced vital capacity, TLC: total lung capacity, % pred: percentage of predicted, CT: computed tomography, SI: sphericity index.

No of patients, n	Total	Fibrosis	Emphysema	CLAD	p
	n = 31	n = 20 (64)	n = 7 (23)	n = 4 (13)	
Age (year)	61 [57; 65]	61 [58; 66]	61 [56; 61]	62 [61; 62]	0.59
Sex (female), n (%)	8 (26)	5 (25)	1 (14)	2 (50)	0.42
Height (cm)	169 ± 10	169 ± 10	171 ± 13	162 ± 14	0.35
Weight (kg)	67 ± 11	69 ± 13	66 ± 14	63 ± 9	0.49
Body mass index (kg/m2)	23.8 ± 3.0	24.0 ± 3.6	22.3 ± 2.7	24.9 ± 4.3	0.32
Side of SLT					
*Right, n* (*%*)	23 (74)	17 (85)	2 (29)	4 (100)	0.006
*Left, n* (*%*)	8 (26)	3 (15)	5 (71)	0 (0)	
Serum albumin level (g/L)	37.2 ± 2.9	37.8 ± 2.8	39.5 ± 4.9	38.0 ± 1.3	0.25
Systemic corticosteroids	15 (48%)	11 (55%)	1 (17%)	3 (75%)	0.14
*Dosing* (*mg*)	13 [7; 20]	15 [10; 20]	20 [20; 20]	5 [5; 5]	0.045
Lung function test					
*FEV1* (*L*)	1.26 ± 0.59	1.49 ± 0.63	0.98 ± 0.60	0.61 ± 0.25	0.005
*FEV1* (% pred)	42 ± 17	50 ± 19	30 ± 13	23 ± 3	0.002
*FVC* (*L*)	2.09 ± 1.00	1.72 ± 0.78	3.23 ± 1.27	1.90 ± 1.19	0.01
*FVC* (*% pred*)	55 ± 21	45 ± 17	80 ± 21	57 ± 20	0.002
*FEV1/FVC ratio* (*%*)	80 [36; 91]	86 [81; 94]	28 [25; 34]	36 [33; 41]	<0.001
*TLC* (*L*)	3.5 [2.9; 4.1]	3.4 [2.6; 3.7]	8.0 [7.5; 8.5]	6.4 [6.0; 6.9]	0.01
*TLC* (*% pred*)	52 [46; 68]	50 [43; 57]	156 [144; 169]	118 [113; 124]	0.01
Six minutes walking test (m)	356 [297; 446]	356 [314; 445]	360 [300; 442]	294 [237; 374]	0.69
Duration of mechanical ventilation (days)	2 [1; 23]	3 [1; 31]	1 [1; 2]	1 [1; 1]	0.99
Duration of intensive care unit stay (days)	12 [5; 28]	13 [6; 35]	7 [3; 11]	14 [10; 19]	0.48
Duration of hospitalisation (days)	37 [28; 71]	48 [30; 78]	37 [27; 65]	33 [31; 44]	0.64
Time between first CT and SLTx (days)	−121 [-69; −83]	−114 [-136; −44]	−121 [-186; −84]	−162 [-197; −123]	0.40
Time between SLTx and second CT (days)	373 [329; 587]	357 [290; 704]	448 [399; 608]	335 [254; 356]	0.07
Tracheal SI	0.76 [0.66; 0.92]	0.82 [0.69; 0.96]	0.86 [0.61; 0.96]	0.42 [0.38; 0.51]	0.05
*Coronal axis* (*cm*)	1.92 [1.70; 2.16]	2.03 [1.82; 2.27]	1.72 [1.44; 2.15]	1.50 [1.24; 1.77]	0.04
*Sagittal axis* (*cm*)	2.20 [1.96; 2.48]	2.25 [1.90; 2.47]	2.20 [1.93; 2.37]	2.06 [1.97; 2.35]	0.95

### 3.2 Principal study outcomes

Principal study outcomes are presented in Table 2. Change in diaphragmatic morphology after SLTx was significantly different between the transplanted and native sides. We noticed a significant increase in Celiac min on the side of the transplanted lung, with an estimated difference of +1.25 mm, *p* = <0.001, while no significant difference was observed in Celiac min on the side of the native lung. Similarly, Mid L1 diaphragmatic thickness of the transplanted lung significantly increased from +4.20 mm to +5.10 mm (+0.90mm, *p* < 0.001), whereas Mid L1 of the native lung remains stable (+0.01 mm, *p* = 0.89). Finally, Diaph height significantly reduced after SLTx on the transplanted side (−1.20 cm, *p* = 0.05), while no change on the native side (+0.02 cm, *p* = 0.88).

**TABLE 2:** Principal study outcomes. SLTx: single-lung transplantation, IQR: interquartile range.

Diaphragmatic measurements	Before SLTx		After SLTx		Wilcoxon signed-rank test		
	Median	IQR	Median	IQR	Difference	CI 95%	p
Celiac min (mm)							
*Transplanted side*	2.90	(2.25; 3.80)	3.60	(2.62; 4.97)	+1.25	(0.70; 1.75)	<0.001
*Native side*	3.00	(2.25; 3.65)	2.80	(2.12; 3.45)	−0.25	(-0.60; 0.15)	0.27
Mid L1 (mm)							
*Transplanted side*	4.20	(3.40; 5.40)	5.10	(3.65; 6.75)	+0.90	(0.45; 1.30)	<0.001
*Native side*	3.90	(3.25; 5.30)	4.10	(3.25; 5.35)	+0.01	(-0.35; 0.45)	0.89
Diaph height (cm)							
*Transplanted side*	7.40	(6.20; 11.00)	6.65	(5.55; 8.67)	−1.20	(-2.55; −0.01)	0.05
*Native side*	6.90	(5.30; 9.40)	7.45	(5.30; 9.67)	+0.02	(-0.45; 0.75)	0.88

**FIGURE 1:**
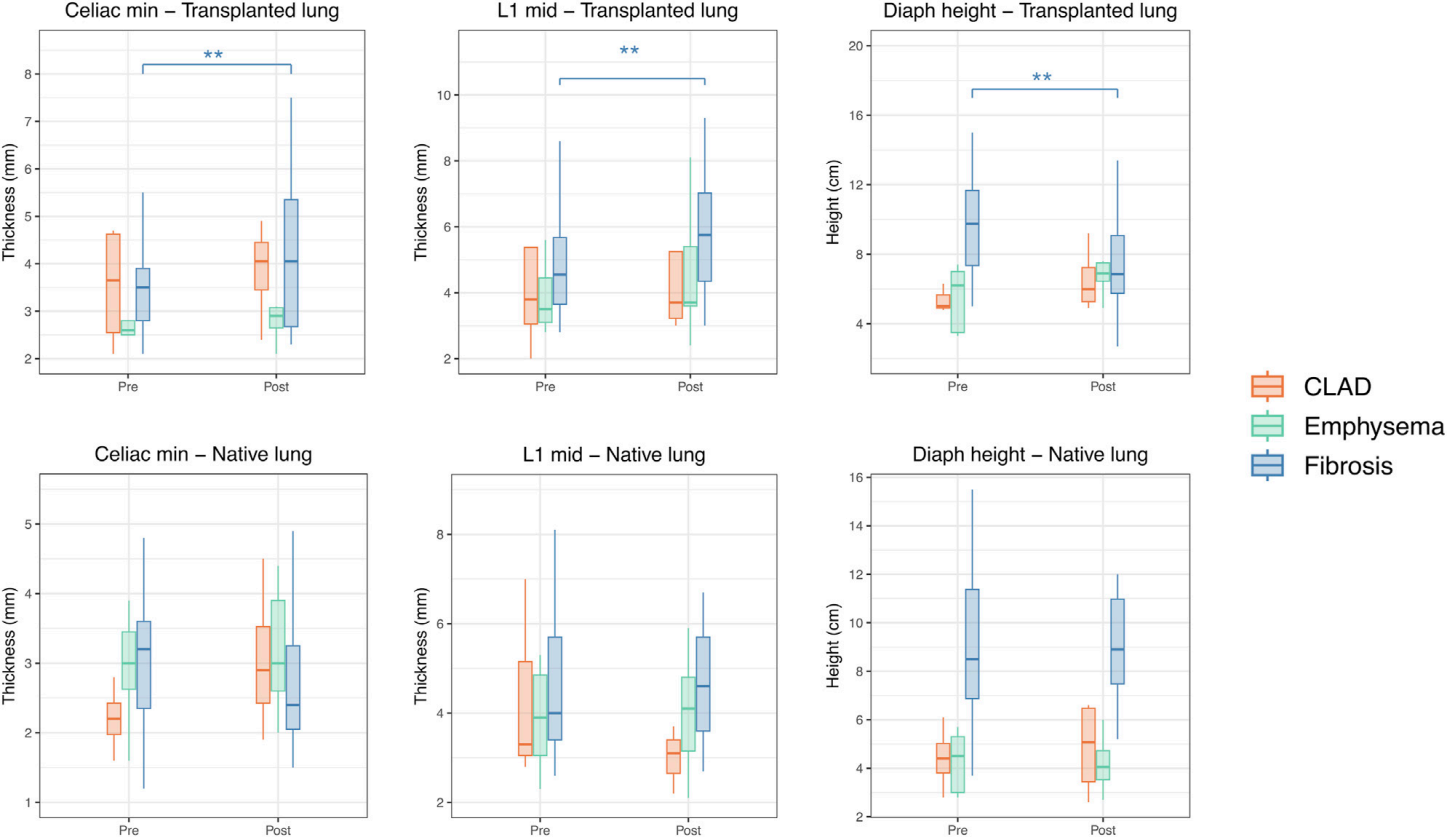
Change in diaphragmatic morphology after SLTx according to the underlying lung disease. Each significance level is associated to a symbol: ** for *p* < 0.01.

Figure 1 represents the change in diaphragmatic morphology according to the underlying diseases. Differences between pre- and post-transplant were found only for fibrotic recipients (*p* < 0.01 for celiac min, L1 mid, and diaph height respectively). Fibrotic recipients had significantly higher diaphragmatic dome in pre-transplantation on both sides (*p* < 0.001 on the transplanted and native sides respectively).

Supplementary Figure S2 represents the change in diaphragmatic morphology on both transplanted and native lungs according to right and left lung transplantation. Celiac min and L1 mid of transplanted lung significantly increased after transplantation, whatever of the side of transplantation (*p* = 0.01 and *p* = 0.006 for right Celiac min and L1 mid respectively, *p* = 0.04 and *p* = 0.004 for left Celiac min and L1 mid, respectively). Diaph height of the transplanted lung significantly decreased when SLTx was performed on the right lung (*p* = 0.02) but not on the left lung (*p* = 0.73). For the native lung, whether right or left, no change in diaphragmatic morphology after SLTx was statistically significant.

### 3.3 Secondary study outcomes

Clinical data such as weight and body mass index, as well as serum albumin level and 6 MWT, did not change significantly after SLTx. However, lung function tests (FEV1 and FVC) improved significantly (Supplementary Table S1).

**FIGURE 2:**
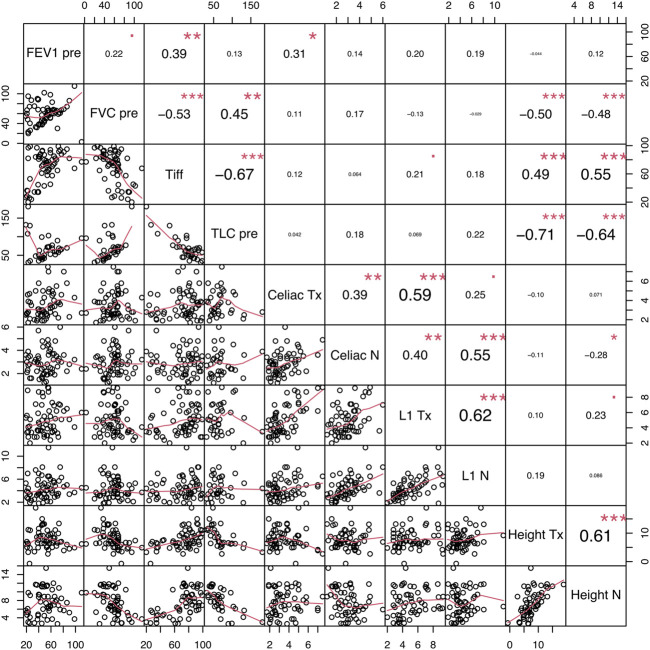
Spearman’s correlation matrix between functional and morphologic parameters. FEV1: forced expiratory volume in the first second, FVC: forced vital capacity, TLC: total lung capacity, Tx: transplanted side, Native: native side. Each significance level is associated to a symbol: *** for *p* < 0.001, ** for *p* < 0.01, * for *p* < 0.05, square dot for *p* < 0.10.

We studied the correlation between lung function tests and the diaphragmatic morphology on CT. Results are presented with a Spearman correlation matrix (Figure 2). Diaph height, whether of the transplanted or native lung, is significantly negatively correlated with FVC and TLC, with a moderate to a strong association (from −0.48 to −0.71, *p* < 0.001). Similarly, Diaph height is correlated with FEV1/FVC ratio, with a moderate association, equally on the transplanted or native side (+0.49 and +0.55, *p* < 0.001, respectively). No significant correlations were found with Celiac min or L1 mid and lung function tests.

## 4 Discussion

In this study, we observed that the diaphragm of the transplanted lung significantly improved after surgery, becoming thicker and less elevated, returning to its anatomical morphology. In contrast, the diaphragm of the native lung remains thinned and at the same level. These changes concerned mainly fibrotic recipients that had severe restriction before LTx. One can hypothesize that a diseased lung may negatively influence the structure of the diaphragm and thus its function. Morphological alterations are potentially reversible, at least partially, as demonstrated by improvements that were observed after the transplantation of a healthy lung.

Diaphragm changes have been observed on both sides of transplantation, although on the left lung morphological improvements were less pronounced. Particularly, Diaph height of the transplanted left lung did not change, as for the right transplanted lung. This can be explained because the left hemidiaphragm is thinner and shorter than the right one. In addition, most patients undergoing left SLTx were transplanted for emphysema, whereas patients undergoing right SLTx were mostly transplanted for pulmonary fibrosis. The nature of the lung disease could have an influence on the structure of its diaphragm. Compared to pulmonary fibrosis, the distension associated with the emphysema may be more damaging to the diaphragm, making it more difficult to return to its normal morphology in the case of LTx. Especially in the context of emphysema being a chronic disease that evolved for years compared to pulmonary fibrosis which may have a more rapid progression. Conversely, fibrotic recipients have severe restriction before transplantation that is associated with a strong elevation of the diaphragmatic dome. Thus, changes after transplantation might be more pronounced compared to emphysema patients and explain why there are differences according to the side of transplantation.

This study also showed some significant correlations between diaphragm morphology and lung function tests. Indeed, we found that Diaph height was correlated with FEV1/FVC ratio and negatively correlated with FVC and TLC. The restriction is classically associated with an elevation of the diaphragm and distension with the flattening of the dome.

Given the preponderant role of the diaphragm in breathing, the search for diaphragmatic impairment when identifying a respiratory disorder is essential. Several studies have investigated the place of CT in the evaluation of diaphragmatic morphology and are interested in the correlation between morphological damage and functional/clinical outcomes. In COPD, compared with the control group, the length and surface of the diaphragm seem to reduce, particularly in the part of the zone of apposition (Tian et al., 2013). Diaphragm density on CT was associated with COPD severity among sixty-five patients (Donovan et al., 2021). In late-onset Pompe disease, early recognition of respiratory insufficiency by assessing diaphragm atrophy on CT could allow an early start of enzyme replacement therapy (Gaeta et al., 2013). In critically ill patients, compared with a control group, the diaphragm thickness was not different upon intensive care unit admission, but decreased during the stay, especially in sepsis (Jung et al., 2014). Similarly, in thirteen mechanically ventilated patients, mean diaphragm thickness reduction was approximately 10% during intensive care unit stay (Lee et al., 2016). Thus, quantifying diaphragm morphology changes on CT after LTx could be an interesting tool to assess outcomes and guide respiratory care such as rehabilitation.

In lung transplantation, only one study assessed diaphragmatic morphology by CT, comparing nine patients with single lung transplantation for emphysema to nine healthy subjects. Using three-dimensional reconstruction of the diaphragm, the total diaphragm surface area and surface area of the dome were smaller in transplanted patients (Cassart et al., 1999). Other methods of evaluating the diaphragm in LTx were assessed. Ultrasound that does not involve patient transportation or exposure to ionizing radiation, provides dynamic assessment of the diaphragm and is an interesting tool to assess diaphragm function. In heart or lung transplant recipients, the presence of diaphragmatic dysfunction on ultrasound was associated with higher pneumonia during hospitalization (Dorffner et al., 1997) and with more days of mechanical ventilation and intensive care unit stay (Ferdinande et al., 2004). In bilateral lung transplantation, patients with permanently elevated diaphragm on chest-X-rays experienced worse lung function tests (Draeger et al., 2021).

The main strength of this work is that we compare by CT the change in diaphragmatic morphology after a SLTx, comparing the transplanted to the native side, taking the patient as his own control. We have provided further evidence that a diseased lung may negatively influence the structure of the diaphragm. However, several limitations can be raised. Because of the retrospective nature of this work, approximately 25% of patients undergoing SLTx during the study period could not be included, due to loss of data. Particularly, many CT could not be evaluated because the diaphragm was not seen completely or because images were too blurred to be analyzed accurately, showing the difficulty of CT assessment of diaphragmatic morphology in daily practice.

## 5 Conclusion

After a SLTx, diaphragmatic morphology significantly changed on the transplanted lung, while remaining altered on the native lung. This highlights that an impaired lung may have a negative impact on its diaphragm. Replacement with a healthy lung can promote the recovery of the diaphragm to its anatomical morphology, reinforcing the close relationship between these two organs. Computed tomography is an interesting tool to assess the diaphragm in the context of lung disease. Further research is needed to determine if anatomical changes in the diaphragm are associated with clinical outcomes.

## Data Availability

The raw data supporting the conclusion of this article will be made available by the authors, without undue reservation.
