# Supplementary material for: Change in diaphragmatic morphology in single-lung transplant recipients: a computed tomographic study
Source: Front Physiol. 2023 Sep 26;14:1220463. doi: 10.3389/fphys.2023.1220463 (PMC10562565; doi:10.3389/fphys.2023.1220463)
Supplement: Supplementary file 1 [file Table1.DOCX]

Supplementary Material

Change in diaphragmatic morphology in single-lung transplant recipients: a computed tomographic study

**François Touchon; Julien Bermudez; Paul Habert; Fabienne Bregeon; Pascal Alexandre Thomas; Martine Reynaud-Gaubert; Benjamin Coiffard.**

*** Correspondence:** Dr Benjamin COIFFARD, Assistance Publique - Hôpitaux de Marseille (AP-HM), Hôpital Nord, Department of Respiratory Medicine and Lung Transplantation, Chemin des Bourrely, 13015 Marseille, France. Phone: (+33) 4 91 96 61 45; Fax: (+33) 4 91 96 61 48. E-mail: bcoiffard.aphm@gmail.com. ORCID: 0000-0002-8896-5346.

# Supplementary Table

**Table S1. Evolution of clinical, biological, and functional data after SLTx.** SLTx: single-lung transplantation, FEV1: forced expiratory volume in the first second, % pred: percentage of predicted, FVC: forced vital capacity, TLC: total lung capacity, 6 MWT: six minutes walking test.

|  |  | **Wilcoxon signed-rank test** | | |
| --- | --- | --- | --- | --- |
|  | Total | Difference | CI 95% | p |
| No of patients, n | 31 |  |  |  |
| Weight (kg) | 67 ± 11 | -0.01 | (-3.50; 2.50) | 0.96 |
| Body mass index (kg/m2) | 23.5 ± 2.9 | -0.20 | (-1.3; 0.80) | 0.58 |
| Post-SLTx corticosteroids | 6 (21) |  |  |  |
| *Dose (mg)* | 6 [3; 15] |  |  |  |
| *Bolus, n (%)* | 11 (39) |  |  |  |
| Serum Albumin level (g/L) | 38.1 ± 4.7 | +0.85 | (-1.7; 2.85) | 0.60 |
| Lung function test |  |  |  |  |
| *FEV1* (*L*) | 1.73 ± 0.58 | +0.45 | (0.26; 0.67) | <0,001 |
| *FEV1* (*% pred*) | 56 [48; 68] | 17 | (10; 25) | <0,001 |
| *FVC* (*L*) | 2.56 ± 0.86 | +0.48 | (0.27; 0.68) | 0,001 |
| *FVC* (*% pred*) | 66 [56; 70] | 11 | (2.5; 18) | 0,01 |
| *FEV1/FVC ratio* (*%*) | 72 [64; 79] | +1.0 | (-8.3; 9.7) | 0,76 |
| *TLC* (*L*) | 4.17 [3.16; 4.75] | +0.59 | (-0.05; 1.25) | 0,06 |
| *TLC* (*% pred*) | 70 [63; 77] | +9.5 | (-3; 23) | 0,12 |
| Six minutes walking test (m) | 400 [341; 479] | 44 | (-8; 108) | 0,11 |

# Supplementary Figures

**Figure S1. Measurements of the diaphragm performed on CT scan.** **A**, Measurement of Celiac min on CT scan. Minimal diaphragm thicknesses (red lines) at the level of the origin of the celiac artery (red arrow). **B**, Measurement of Mid L1 on CT scan. Diaphragm thicknesses (red lines) along the anterior part of the L1 vertebral body at mid-level (red arrow). **C**, Measurement of Diaph height on CT scan. Heights (red lines) from the highest point of the diaphragmatic dome to its perpendicular intersection with a line (white line) through the upper pole of L1 (red arrow).

**Figure S2. Change in diaphragmatic morphology after SLTx according to right and left lung transplantation.** Each significance level is associated to a symbol: ** for p<0.01, * for p<0.05.
