# Supplementary material for: Change in diaphragmatic morphology in single-lung transplant recipients: a computed tomographic study
Source: Front Physiol. 2023 Sep 26;14:1220463. doi: 10.3389/fphys.2023.1220463 (PMC10562565; doi:10.3389/fphys.2023.1220463)
Supplement: Supplementary file 2 [file Image2.PDF]

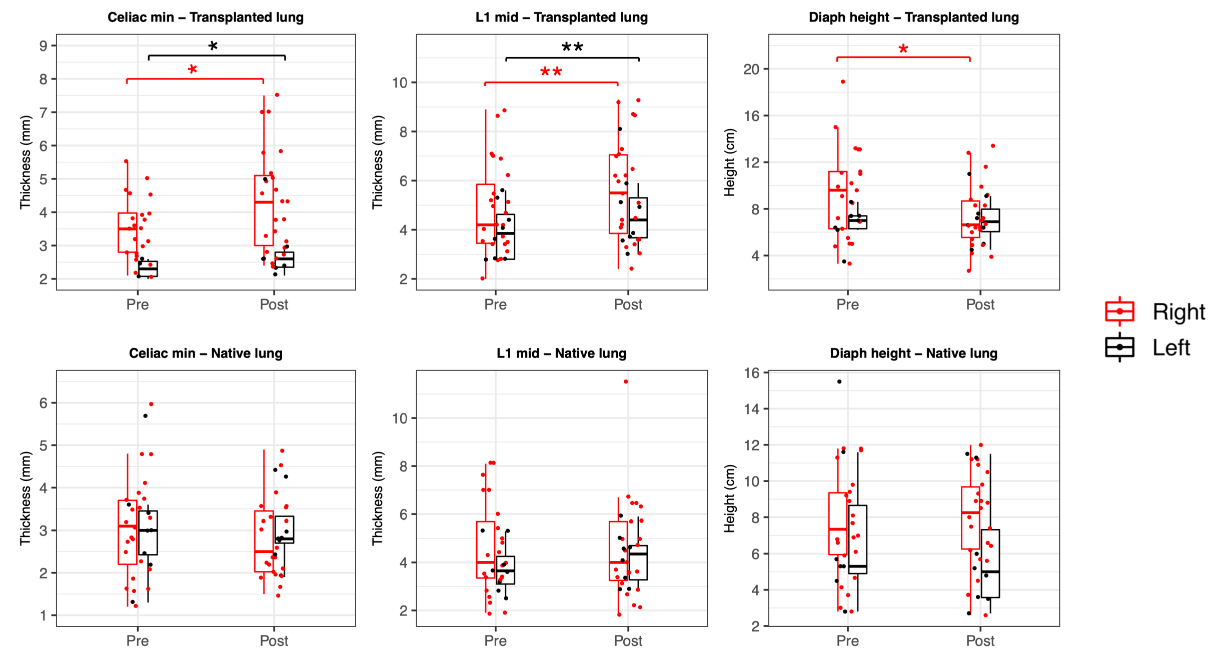

Celiac min – Transplanted lung
L1 mid – Transplanted lung
Diaph height – Transplanted lung
Celiac min – Native lung
L1 mid – Native lung
Diaph height – Native lung
Thickness (mm)
Height (cm)
Pre
Post
*
**
Right
Left
