# Supplementary figures and images for: Change in diaphragmatic morphology in single-lung transplant recipients: a computed tomographic study
Source: Front Physiol. 2023 Sep 26;14:1220463. doi: 10.3389/fphys.2023.1220463 (PMC10562565; doi:10.3389/fphys.2023.1220463)

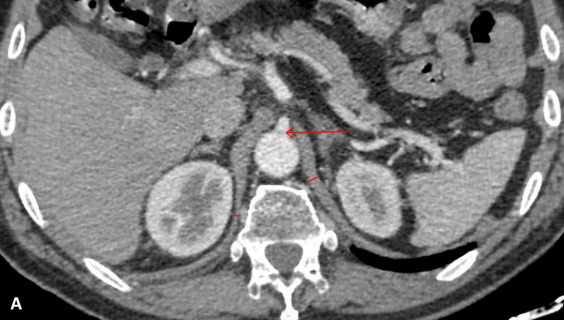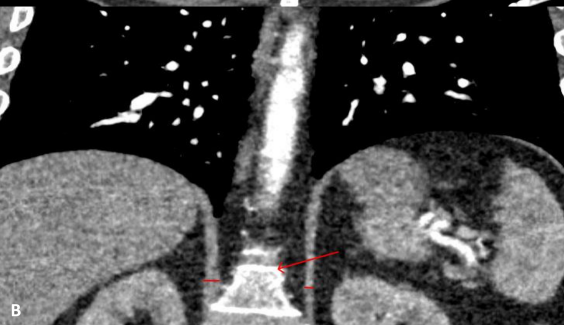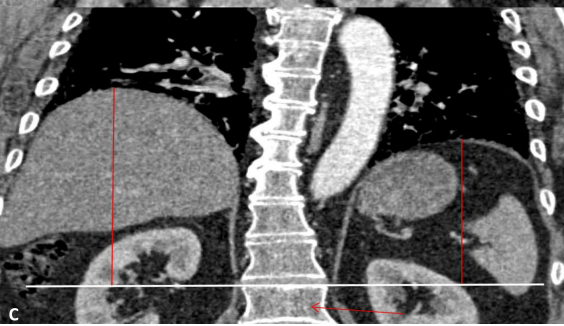

Supplement: Supplementary file 3 [file Image1.PDF]
